# Desmoplastic Small Round-Cell Tumor in a Young Indigenous Australian Man: A Case Report

**DOI:** 10.1200/JGO.2016.006163

**Published:** 2016-08-17

**Authors:** Sabeeh-ur-Rehman Butt, James Martin Horsely Bull, Amy Scott

**Affiliations:** All authors, Lismore Base Hospital, Lismore, Australia.

## INTRODUCTION

Desmoplastic small round-cell tumor (DSRCT) is a rare and highly aggressive neoplasm of children and young adults that primarily arises from the abdomen with widespread peritoneal involvement and is characterized by distinct clinicopathologic features.^1^ Therapeutic management of DSRCT remains challenging, and no standard therapy is currently described in the literature. Despite the combination of aggressive treatments such as chemotherapy, debulking surgery, and whole abdominal radiation, the prognosis of DSRCT remains extremely poor.^2^ There are fewer than 200 cases reported in literature. We report the first case of an Australian-indigenous man with DSRCT and also describe our treatment approach for this rare cancer.

## CASE REPORT

A 20-year-old indigenous Australian man was admitted to the hospital with a 3-week history of progressive abdominal discomfort, lethargy, and weight loss, associated with nausea and occasional night sweats but no urinary or bowel symptoms. His medical history was significant for recurrent tonsillar infections, intravenous use of amphetamines, and marijuana smoking. He was not on any regular medications, and there was no family history of cancer. Clinical examination revealed a young man with a low body mass index. He was afebrile and hemodynamically stable. On abdominal examination, a firm mass was palpable in the lower abdominal region, and there was palpable inguinal lymphadenopathy, while rectal examination revealed a smooth, firm, nontender mass. Laboratory results showed mild, normocytic, normochromic anemia, with a hemoglobin level of 124 g/L and a normal white blood cell count. Results also showed acute kidney injury, with a creatinine level of 227 μmol/L, whereas liver function tests revealed a normal bilirubin level and an alkaline phosphatase level of 241 U/L, with an AST level of 66 U/L and an ALT level of 27 U/L. His inflammatory markers were within the normal range.

**Figure 1:**
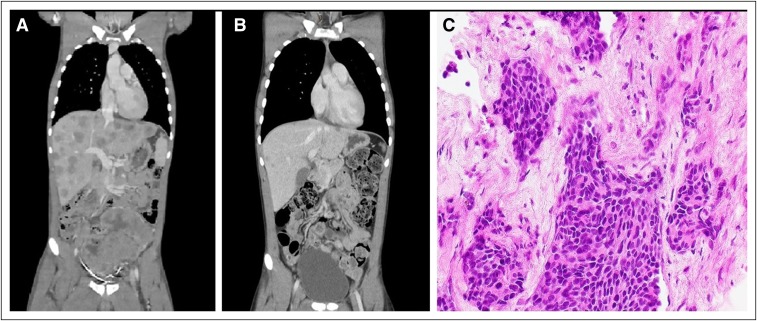
Computed tomography (CT) scan showing a large soft-tissue mass in the pelvis and multiple, hypodense lesions in the liver. (A) CT scan showing a large tumor in abdomen with hepatic metastasis before chemotherapy. (B) Markedly reduced tumor burden after three cycles of chemotherapy. (C) Characteristic desmoplastic small round cells with scanty fibrous stroma.

A computed tomography (CT) scan showed a large soft-tissue mass in the pelvis and multiple, hypodense lesions in the liver, along with porta hepatis and retroperitoneal and external iliac chain lymphadenopathy (Fig 1A). There were multiple pelvic intraperitoneal metastases and mild to moderate bilateral hydronephrosis. There was no evidence of thoracic or cerebral involvement. An initial histopathology from a CT-guided biopsy of a retroperitoneal lymph node showed irregular clusters and geographic-shaped sheets of neoplastic small cells with a high nuclear-to-cytoplasmic ratio and some degree of necrosis supported by fibrous stroma, which was reported to be a variant of small cell cancer (Fig 1C). The patient was commenced on cisplatin and etoposide. However, subsequent immunohistochemical staining showed positive results for AE1/AE3, desmin, vimentin, cytokeratin, and epithelial membrane antigen. Interestingly, *EWSR-1* rearrangement was also detected; however, *WT-1* results were negative. On the basis of this information, a final diagnosis of DSRCT was made.

The patient’s renal function improved with intravenous hydration alone, and he showed clinical signs of improvement after one cycle of chemotherapy; therefore, the decision was made to continue with the same regimen. A repeated CT scan after three cycles of chemotherapy showed a good measurable response (Fig 1B), and as of this writing, the patient continues to improve. He is scheduled to receive a total of six cycles with the current regimen, and depending on the response, a multidisciplinary approach will be considered thereafter.

## DISCUSSION

DSRCT was first described in 1989 by Gerald and Rosai,^1^ and although only a few cases have been reported in the literature, to our knowledge, it has not been described in the indigenous Australian population, making this case report unique. The tumor is composed of dense desmoplastic stroma with a nesting pattern of small, round, blue cellular growth, occasional focal rhabdoid features, and immunohistochemical reactivity for epithelial, muscle, and neural markers. DSRCT is associated with a characteristic chromosomal translocation, t(11;22)(p13;q12), which fuses the N-terminus of the Ewing sarcoma (EWS) gene to the C-terminus of the Wilms tumor (*WT-1*) gene.^3^ The presence of this translocation provides confirmation of the diagnosis. The majority of these tumors stain positive for EWS-1, desmin, cytokeratin, vimentin, epithelial membrane antigen, and *WT-1*,^4,5^ which is again consistent with the immunostaining data of our patient, except for *WT-1*.

Patients are typically 5 to 30 years of age at presentation, and 90% are male. They often present with advanced abdominal disease, with large masses and/or extensive seeding into the visceral and parietal peritoneum. Most patients are asymptomatic until they have a large tumor burden, when they may present with abdominal pain or distention along with nausea and vomiting.^6^ This is consistent with the presentation of our patient. Because of the rarity of this tumor, misdiagnosis is common and treatment remains a challenge; despite the use of polychemotherapy and surgery, the overall survival rate is 15% at 5 years.^7^ One case study examined a cyclophosphamide, doxorubicin, and vincristine regimen alternating with ifosfamide and etoposide, which showed a median survival time of 19 months.^8^ But mostly, owing to the rarity of DSRCT, mostly different chemotherapy regimens, including anthracyclines, cisplatin, and etoposide, have been tried with limited success.^9-11^ We used the cisplatin and etoposide regimen before the immunostain results confirmed the diagnosis of DSRCT. However, both of these agents have been used in published studies,^11^ and our patient is currently responding to treatment, as evidenced by the follow-up imaging, leaving other polychemotherapy options, including anthracyclines, to be explored in the future.

In conclusion, DSRCT is a rare abdominal tumor in young men that is highly likely to be misdiagnosed, despite its well-defined clinicopathologic properties. Because of its aggressive nature, it is associated with a poor outcome, despite polychemotherapy regimens. The diagnosis should therefore be considered in young men, even in indigenous Australian populations, with a multifocal peritoneal soft tissue mass that is usually metastatic at the time of diagnosis, because early detection may influence management options, thereby improving survival.
